# Development and validation of an interpretable machine learning model for predicting early acute kidney injury and altered consciousness in cardiac surgery patients admitted to the intensive care unit: a retrospective study

**DOI:** 10.3389/fendo.2026.1959304

**Published:** 2026-10-07

**Authors:** Shu Yang, Jing Luo, Lianzheng Ma, Dongxiao Huang, Minmin Zhu, Linlin Cai

**Affiliations:** 1 Department of Anesthesiology and Pain Medicine, Wuxi No.2 People’s Hospital (Jiangnan University Medical Center), Wuxi, China; 2 Wuxi School of Medicine, Jiangnan University, Wuxi, China; 3 Department of Anesthesiology, Affiliated Obstetrics and Gynecology Hospital of Jiangnan University, Wuxi, China

**Keywords:** acute kidney injury, altered consciousness, cardiac surgery, intensive care unit, machine learning

## Abstract

**Background:**

Acute kidney injury (AKI) and altered consciousness are common and serious complications following cardiac surgery, both associated with increased morbidity and mortality in the intensive care unit (ICU). Early prediction of these complications is essential for timely intervention. We aimed to develop and validate an interpretable machine learning model for predicting early AKI and altered consciousness risk in this population.

**Methods:**

We retrospectively extracted data from the MIMIC-IV database. Adult patients who underwent cardiac surgery within 24 h before ICU admission were included. The primary outcome was early AKI defined by KDIGO criteria within 48 h after ICU admission. The secondary outcome was altered consciousness within 24 h after ICU admission, defined as a Glasgow Coma Scale (GCS) score ≤12. A comprehensive set of candidate variables was screened using four feature selection methods (LassoCV, mRMR, mutual information, and *F*-test) with a voting strategy. Eight machine learning models were developed and compared. SHapley Additive exPlanations (SHAP) values were used to interpret the best-performing model. External validation was performed using data from cardiac surgery patients at Wuxi No.2 People’s Hospital between 1 January 2020 and 31 December 2025.

**Result:**

A total of 5,288 patients were included, with an AKI incidence of 76.2% (4,031/5,288) and an altered consciousness incidence of 22.8% (1,206/5,288). For the primary outcome of AKI, the XGBoost model achieved a training AUC of 0.787 (95% CI: 0.770–0.804) and an internal test AUC of 0.659 (95% CI: 0.629–0.688). For the secondary outcome of altered consciousness, the XGBoost model achieved an internal test AUC of 0.951 (95% CI: 0.936–0.963). Calibration curve and decision curve analysis confirmed good calibration and clinical utility for both models. SHAP analysis identified SpO_2_, age, APSIII score, creatinine, and white blood cell count as the top 5 most important predictors for AKI. External validation was performed in an independent cohort of 395 cardiac surgery ICU patients from Wuxi No.2 People’s Hospital, in whom the incidence of AKI and altered consciousness was 28.1% and 67.3%, respectively. The models retained acceptable discrimination in this external cohort (AKI: AUC 0.764, 95% CI: 0.711–0.818; altered consciousness: AUC 0.679, 95% CI: 0.615–0.737).

**Conclusion:**

We developed interpretable XGBoost-based prediction models for both early AKI and altered consciousness risk in cardiac surgery patients admitted to the ICU. The models exhibited excellent discrimination, calibration, and clinical utility, which may facilitate early identification of high-risk patients and guide personalized interventions.

## 1 Introduction

Acute kidney injury (AKI) is one of the most frequent and severe post-cardiac surgery complications, which may affect up to 5%–42% of patients based on the definition and type of surgery performed (1, 2). The association between surgery-associated AKI (CSA-AKI) and longer length of stay, higher healthcare costs, and dramatically increased in-hospital and long-term mortality has been reported (3, 4). Although perioperative care has improved, the rate of CSA-AKI remains high, especially in patients who need intensive care unit (ICU) treatment after surgery (5).

Altered consciousness is also a common complication in the early postoperative stage after cardiac surgery. Patients who are admitted to the ICU can develop delirium, impaired cognition as a result of sedation, or other disorders of consciousness because of such causes as hypoperfusion, inflammation, metabolic disturbance, and drug effects of anesthesia. The clinical significance of early detection of patients at risk of altered consciousness includes the need to change sedation procedures, neurological monitoring, and hemodynamic control (6).

The identification of high-risk patients with AKI is essential because it allows introducing timely preventative interventions, like optimizing hemodynamics, eliminating nephrotoxins, and starting renal replacement therapy if necessary (6, 7). Conventional risk prediction models based on the Cleveland Clinic score and the Simplified Renal Index are based on a small number of preoperative variables and might not be able to fully account for the intricate, non-linear relationships between several perioperative indicators (8, 9). Over the past few years, machine learning (ML) algorithms have been drawing more and more attention in medical prognosis because they are capable of modeling high-dimensional data and revealing underlying trends without making any assumptions about the relationships between the variables (10, 11).

The Medical Information Mart for Intensive Care (MIMIC) database is a large-scale and publicly accessible critical care database that has become popular in developing clinical prediction models (12, 13). Many research studies have used MIMIC to develop AKI prediction models in different populations, but there are only a few studies that have focused on the population of cardiac surgery patients who were admitted to the ICU with a wide range of pre-ICU variables and systematic comparison with ML models (14, 15). Also, interpretability of ML models is an important issue in clinical practice since clinicians require understanding the reason behind the predictions to provide informed choices (16).

In summary, the current research has attempted to create and test an interpretable ML-based prediction model of early AKI and altered consciousness risk in patients undergoing cardiac surgery who are admitted to the ICU with data from the MIMIC-IV database. To compare several ML algorithms systematically, we used a powerful feature selection approach combining four methods and SHAP values to improve model transparency and clinical usefulness.

## 2 Methods

### 2.1 Study design and data source

The present study was based on the MIMIC-IV (version 2.2) database that has de-identified health data of patients who were admitted to Beth Israel Deaconess Medical Center during 2008–2022 (12). The database has detailed clinical data, including demographics, vital signs, laboratory findings, medications, interventions, and diagnoses. As the database is open to the public and all patient identifiers are de-identified, the necessity of obtaining informed consent and ethical clearance was not required. The Transparent Reporting of a Multivariable Prediction Model in Individual Prognosis or Diagnosis (TRIPOD) guideline (17) was used in this study. The author, Yang Shu, has completed the Collaborative Institutional Training Initiative (CITI) program (Credential Number: 62274870).

### 2.2 Study population

Adult patients (age ≥ 18 years) who had undergone cardiac surgery not more than 24 h prior to their first ICU admission during hospitalization were taken into consideration. Exclusion criteria were used to include patients in the study, namely, (1) ICU stay less than 24 h; (2) death before ICU admission; (3) lack of complete records of the outcome assessment; (4) no GCS scores; or (5) absence of important laboratory or vital sign information, defined as (i) no serum creatinine measurement available within the first 24 h after ICU admission (precluding KDIGO assessment), or (ii) missing values in more than 30% of the core candidate variables (complete blood count, electrolytes, renal function, coagulation profile, lactate, blood gas parameters, and routinely monitored vital signs) within the first 24 h after ICU admission. **Figure 1** shows the detailed patient selection flowchart, which now also depicts the selection of the external validation cohort.

**Figure 1:**
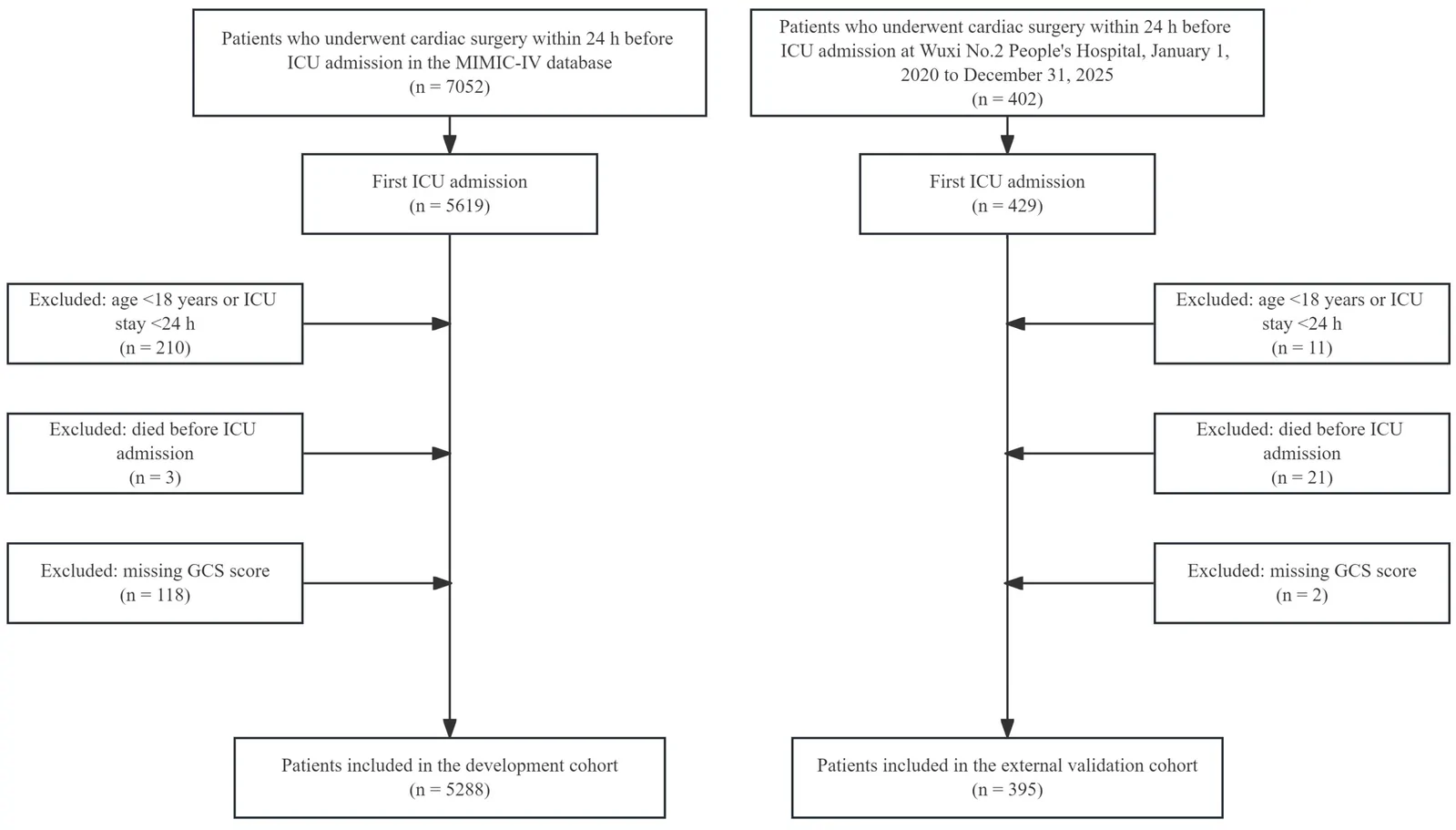
Study flowchart of patient selection for the development cohort and the external validation cohort.

### 2.3 Variable selection and definition

The entire list of candidate predictor variables was derived with the help of the MIMIC-IV database, which includes demographic variables (age and gender), type of surgery (CABG, valve surgery, aortic surgery, combined surgery), comorbidities (diabetes, congestive heart failure, renal disease, myocardial infarction, cerebrovascular disease, peripheral vascular disease, chronic pulmonary disease, mild liver disease, malignant cancer, paraplegia, dementia, rheumatic disease, peptic ulcer disease), medications (aspirin, heparin, statin, sedation), clinical interventions (mechanical ventilation), vital signs (heart rate, systolic blood pressure, diastolic blood pressure, mean blood pressure, respiratory rate, temperature, SpO_2_), and laboratory parameters (hematocrit, hemoglobin, platelets, white blood cell count, anion gap, bicarbonate, blood urea nitrogen, chloride, creatinine, glucose, sodium, potassium, international normalized ratio, prothrombin time, partial thromboplastin time, lactate, pH). Another composite severity score that was used is called the Acute Physiology Score III (APSIII). The continuous variables were measured in the form of mean values during the initial 24 h after ICU admission or as the latest preoperative values 24 h prior to surgery. The missing values were addressed by applying the k-nearest neighbors (KNN) imputation technique. Pre-imputation missingness rates for all candidate variables were tabulated for both cohorts (**Supplementary Table S1**). In the MIMIC-IV cohort, missingness was low (maximum 16.5% for temperature; ≤2.3% for all other modeling variables), and a complete-case sensitivity analysis (*n* = 5,229, 98.9% of the cohort) yielded consistent discrimination (test AUC 0.682 for AKI and 0.939 for altered consciousness). The external validation cohort contained no missing values for any variable; therefore, no imputation was required for the external cohort, and the external validation analyses are complete data analyses by construction.

### 2.4 Outcome definitions

The main finding was the occurrence of early AKI within 48 h of ICU admission. AKI was defined using the Kidney Disease: Improving Global Outcomes (KDIGO) guidelines depending on serum creatinine fluctuations: an elevation in serum creatinine of at least 0.3 mg/dL over 48 h or at least 1.5-fold the baseline level in 7 days (18). Creatinine baseline was determined as the minimum creatinine measurement recorded in the last 7 days prior to ICU admission. Sensitivity analyses using alternative baseline definitions (first ICU creatinine; CKD-EPI back-calculation assuming an eGFR of 75 mL/min/1.73 m²) were performed in the external validation cohort, for which the required creatinine components were available. The secondary endpoint was the presence of altered consciousness after 24 h of ICU admission, which was described as a Glasgow Coma Scale (GCS) value of less than or equal to 12. RASS/SAS sedation scores were not systematically available in our MIMIC-IV extract; deeply sedated patients could therefore not be excluded, and sedation exposure within the first 24 h was instead included as a candidate predictor. This outcome captures a decreased level of consciousness—which may be driven by sedation, mechanical ventilation, or neurological injury—rather than delirium per se, and is therefore referred to as “altered consciousness” throughout this manuscript. Participants not satisfying the criteria were categorized as the non-event group in relation to that specific outcome.

### 2.5 Feature selection

We used a multi-algorithm feature selection method to achieve better stability and generalizability of the models. The training group was subjected to four different procedures, namely, (1) LassoCV, which uses L1 regularization with cross-validation to identify sparse features; (2) minimum redundancy maximum relevance (mRMR), which chooses features with high correlation to the outcome and the smallest interfeature redundancy; (3) mutual information, which measures the dependence of each feature on the outcome; and (4) *F*-test, which determines the univariate statistical significance of a given feature. Candidate variables consisting of features chosen by two or more methods (voting threshold of 50%) were kept. A Venn diagram was used to illustrate the overlap between the four methods (**Figure 2**). The 20 variables finally included in model development after clinical relevance review and redundancy removal were age, gender, diabetes, renal disease, congestive heart failure, creatinine, blood urea nitrogen, potassium, bicarbonate, anion gap, lactate, mean blood pressure, SpO_2_, heart rate, mechanical ventilation, APSIII, hemoglobin, white blood cell count, platelets, and sedation. All feature-selection procedures were performed exclusively on the training set (*n* = 3,701); the internal test set and the external validation cohort were not used for variable selection, hyperparameter tuning, or threshold selection, thereby avoiding information leakage and optimistic bias. The robustness of the voting threshold was assessed in a sensitivity analysis in which the consensus threshold was varied from ≥1 to ≥4 votes (**Supplementary Table S2**), and feature-distribution consistency between the training and test sets was verified using standardized mean differences (all < 0.1; **Supplementary Table S3**).

**Figure 2:**
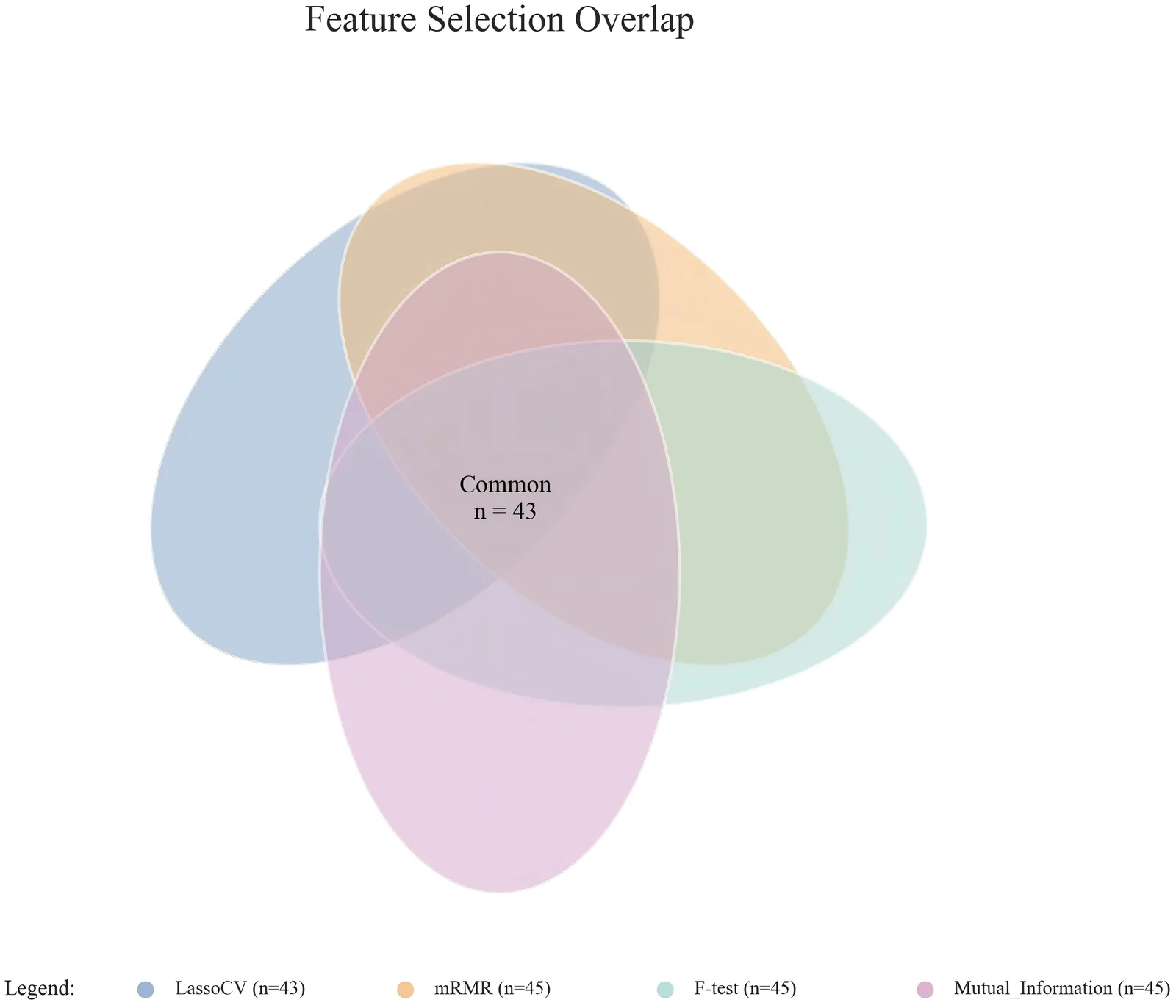
Feature selection overlap among the four methods (LassoCV, mRMR, *F*-test, and mutual information).

### 2.6 Model development and statistical analysis

XGBoost, LightGBM, AdaBoost, support vector machine (SVM), multilayer perceptron (MLP), Gaussian naive Bayes (GNB), decision tree (DT), and gradient boosting decision tree (GBDT) are eight ML algorithms that were implemented and compared. Stratified random sampling was used to divide the dataset into a training set (70%) and a test set (30%) at random. Grid search and hyperparameter tuning was done on the training set through five-fold cross-validation. Area under the receiver operating characteristic curve (AUC) was used to measure model discrimination with a 95% confidence interval (CI). Performance was measured through the precision-recall curve and average precision (AP) in the scenario of class imbalance. Calibration curves and the Brier score were employed to evaluate model calibration. Decision curve analysis (DCA) was used as a measure of clinical utility. The SHAP framework was used to explain the top-scoring model. A two-tailed *P*-value less than 0.05 was statistically significant. No resampling (over- or undersampling) was applied; class weighting was evaluated in a sensitivity analysis by setting the XGBoost scale_pos_weight parameter to the inverse prevalence ratio of the training set. Decision thresholds were derived by maximizing the Youden index on the training set and were then applied unchanged to the internal test set and the external validation cohort; sensitivity, specificity, positive predictive value (PPV), and negative predictive value (NPV) with 95% confidence intervals were reported at these pre-specified thresholds. Pairwise DeLong tests were used to compare the AUC of XGBoost with that of each alternative algorithm. Absolute net benefit was quantified as net true-positive equivalents per 100 patients, and the net reclassification improvement (NRI) and integrated discrimination improvement (IDI) were computed against an APSIII-only logistic regression reference model. All the analyses were performed in Python 3.9 and R 4.3.1.

## 3 Results

### 3.1 Baseline characteristics

There were 7,052 patients that have undergone cardiac surgery in less than 24 h before ICU admission and were extracted from the MIMIC-IV database. Once the inclusion criteria were implemented, 5,288 patients were included in the final analysis (**Figure 1**). Early AKI incidence within 48 h after ICU admission was found to be 76.2% (4,031/5,288), and the incidence of altered consciousness within 24 h after ICU admission was recorded as 22.8% (1,206/5,288). The median age of the cohort was 68.0 (IQR: 60.0–76.0) years, and there was a greater proportion of male patients (71.5%). The most frequent surgical procedure was coronary artery bypass grafting (CABG, 59.1%), then valve surgery (24.3%), combined surgery (14.2%), and aortic surgery (2.4%). The AKI group had significantly older individuals; a higher diabetes rate, congestive heart failure, and renal disease rate; higher levels of creatinine, blood urea nitrogen (BUN), WBC, and potassium; and lower SpO_2_ and hemoglobin levels (all *P* < 0.05) compared with the non-AKI group. The complete baseline characteristics are provided in **Table 1**. Patients with altered consciousness were older than those without altered consciousness [70.0 (61.0–78.0) vs. 68.0 (60.0–76.0) years, *P* = 0.002] and had a lower proportion of male patients (68.0% vs. 72.5%, *P* = 0.002). Renal disease (20.1% vs. 15.7%, *P* < 0.001) and congestive heart failure (31.4% vs. 24.9%, *P* < 0.001) were more prevalent among patients with altered consciousness, whereas the prevalence of diabetes was comparable between groups. Patients with altered consciousness also exhibited higher creatinine, blood urea nitrogen, anion gap, and lactate levels, together with lower hemoglobin and platelet counts. They were more likely to receive mechanical ventilation (15.0% vs. 7.2%, *P* < 0.001) and had substantially higher APS III scores [72.0 (53.0–82.0) vs. 32.0 (25.0–39.0), *P* < 0.001]. In contrast, mean arterial pressure, SpO_2_, white blood cell count, and potassium levels did not differ significantly between the groups (**Supplementary Table S4**). According to the KDIGO staging, stage 1, stage 2, and stage 3 events accounted for 38.4% (1,548/4,031), 54.4% (2,192/4,031), and 7.2% (291/4,031) of AKI events, respectively (**Supplementary Tables S5**, **S6**).

**Table 1:** Comparison of baseline characteristics between the AKI group and the non-AKI group.

Variables	Total (*n* = 5,288)	Non-AKI (*n* = 1,257)	AKI (*n* = 4,031)	*P*
Type of surgery, *n* (%)				< 0.001
CABG	3,123 (59.1)	781 (62.1)	2,342 (58.1)	
Valve surgery	1,286 (24.3)	324 (25.8)	962 (23.9)	
Aortic surgery	127 (2.4)	23 (1.8)	104 (2.6)	
Combined surgery	752 (14.2)	129 (10.3)	623 (15.5)	
Characteristics				
Gender, *n* (%)				0.013
Female	1,509 (28.5)	324 (25.8)	1,185 (29.4)	
Male	3,779 (71.5)	933 (74.2)	2,846 (70.6)	
Age (years), median (IQR)	68.0 (60.0, 76.0)	66.0 (58.0, 74.0)	69.0 (61.0, 77.0)	<0.001
Heart rate (beats/min), median (IQR)	81.2 (76.2, 87.4)	82.1 (76.9, 88.5)	81.0 (76.0, 87.0)	<0.001
SBP (mmHg), median (IQR)	111.8 (106.8, 117.4)	112.8 (107.6, 117.9)	111.5 (106.6, 117.3)	<0.001
DBP (mmHg), median (IQR)	56.6 (52.4, 60.8)	57.9 (53.6, 61.8)	56.2 (51.9, 60.5)	<0.001
MBP (mmHg), median (IQR)	73.8 (70.1, 77.4)	75.0 (71.3, 78.5)	73.4 (69.7, 77.1)	<0.001
Respiratory rate (breaths/min), median (IQR)	17.4 (15.9, 19.0)	17.3 (15.9, 18.9)	17.4 (15.9, 19.1)	0.22
Temperature (°C), median (IQR)	36.7 (36.5, 36.9)	36.7 (36.5, 36.9)	36.7 (36.5, 36.9)	0.198
SpO_2_ (%), median (IQR)	98.0 (97.0, 98.8)	98.1 (97.2, 98.9)	97.9 (97.0, 98.8)	<0.001
Laboratory				
Hematocrit (%), median (IQR)	26.5 (23.8, 29.8)	26.9 (24.2, 30.0)	26.4 (23.6, 29.7)	<0.001
Hemoglobin (g/dL), median (IQR)	9.0 (8.0, 10.1)	9.2 (8.2, 10.2)	9.0 (7.9, 10.1)	<0.001
Platelets (10^9^/L), median (IQR)	129.0 (103.0, 159.0)	128.0 (105.0, 157.0)	129.0 (103.0, 159.0)	0.714
WBC (10^9^/L), median (IQR)	15.4 (12.2, 19.3)	14.9 (11.9, 18.3)	15.6 (12.3, 19.5)	<0.001
Anion gap (mmol/L), median (IQR)	13.0 (11.0, 14.0)	12.0 (11.0, 14.0)	13.0 (11.0, 15.0)	<0.001
Bicarbonate (mmol/L), median (IQR)	22.0 (21.0, 24.0)	23.0 (21.0, 24.0)	22.0 (21.0, 24.0)	<0.001
Bun (mg/dL), median (IQR)	17.0 (14.0, 22.0)	15.0 (12.0, 19.0)	18.0 (14.0, 23.0)	<0.001
Chloride (mmol/L), median (IQR)	109.0 (107.0, 112.0)	110.0 (107.0, 112.0)	109.0 (107.0, 111.0)	0.016
Creatinine (mg/dL), median (IQR)	0.9 (0.8, 1.2)	0.9 (0.7, 1.0)	1.0 (0.8, 1.2)	<0.001
Glucose (mg/dL), median (IQR)	121.0 (107.0, 139.0)	118.0 (104.0, 134.0)	123.0 (108.0, 141.0)	<0.001
Sodium (mmol/L), median (IQR)	137.0 (136.0, 139.0)	137.0 (135.0, 139.0)	137.0 (136.0, 139.0)	<0.001
Potassium (mmol/L), median (IQR)	4.6 (4.3, 4.9)	4.5 (4.3, 4.8)	4.6 (4.4, 4.9)	<0.001
INR, median (IQR)	1.4 (1.3, 1.6)	1.4 (1.3, 1.5)	1.4 (1.3, 1.6)	<0.001
PT (s), median (IQR)	15.5 (14.3, 17.0)	15.4 (14.3, 16.6)	15.6 (14.4, 17.1)	<0.001
PTT (s), median (IQR)	33.8 (29.9, 41.6)	33.3 (29.8, 39.7)	34.0 (29.9, 42.3)	0.015
Lactate (mmol/L), median (IQR)	2.6 (2.0, 3.4)	2.5 (1.9, 3.2)	2.6 (2.0, 3.4)	0.003
PH, median (IQR)	7.3 (7.3, 7.3)	7.3 (7.3, 7.3)	7.3 (7.3, 7.3)	<0.001
Comorbidities				
Myocardial infarction, *n* (%)	1,443 (27.3)	317 (25.2)	1,126 (27.9)	0.059
Congestive heart failure, *n* (%)	1,396 (26.4)	247 (19.6)	1,149 (28.5)	<0.001
Peripheral vascular disease, *n* (%)	918 (17.4)	180 (14.3)	738 (18.3)	0.001
Dementia, *n* (%)	27 (0.5)	4 (0.3)	23 (0.6)	0.273
Cerebrovascular disease, *n* (%)	556 (10.5)	99 (7.9)	457 (11.3)	<0.001
Chronic pulmonary disease, *n* (%)	1,182 (22.4)	250 (19.9)	932 (23.1)	0.016
Rheumatic disease, *n* (%)	160 (3.0)	32 (2.5)	128 (3.2)	0.255
Peptic ulcer disease, *n* (%)	30 (0.6)	8 (0.6)	22 (0.5)	0.709
Mild liver disease, *n* (%)	165 (3.1)	32 (2.5)	133 (3.3)	0.18
Diabetes, *n* (%)	1,890 (35.7)	365 (29)	1,525 (37.8)	<0.001
Paraplegia, *n* (%)	52 (1.0)	7 (0.6)	45 (1.1)	0.079
Renal disease, *n* (%)	882 (16.7)	129 (10.3)	753 (18.7)	<0.001
Malignant cancer, *n* (%)	151 (2.9)	30 (2.4)	121 (3)	0.253
Severity scores.				
Charlson Comorbidity Index, median (IQR)	5.0 (4.0, 6.0)	4.0 (3.0, 6.0)	5.0 (4.0, 7.0)	<0.001
APSIII, median (IQR)	35.0 (27.0, 48.0)	31.0 (24.0, 41.0)	36.0 (28.0, 50.0)	<0.001
SAPSII, median (IQR)	36.0 (29.0, 43.0)	33.0 (27.0, 40.0)	37.0 (30.0, 45.0)	<0.001
OASIS, median (IQR)	32.0 (26.0, 37.0)	30.0 (24.0, 34.0)	32.0 (27.0, 38.0)	<0.001
Treatment				
Aspirin, *n* (%)	3,387 (64.1)	827 (65.8)	2,560 (63.5)	0.141
Heparin, *n* (%)	377 (7.1)	84 (6.7)	293 (7.3)	0.481
Statin, *n* (%)	3,160 (59.8)	763 (60.7)	2,397 (59.5)	0.435
Sedation, *n* (%)	5,123 (96.9)	1,210 (96.3)	3,913 (97.1)	0.148
Mechanical ventilation, *n* (%)	476 (9.0)	90 (7.2)	386 (9.6)	0.009

SBP, systolic blood pressure; DBP, diastolic blood pressure; MBP, mean arterial pressure; INR, international normalized ratio; PT, prothrombin time; PTT, partial thromboplastin time.

### 3.2 Feature selection

The four feature selection methods found 43, 45, 45, and 45 candidate variables, respectively. All four methods chose a common total of 43 variables, which is an indication that there was a high level of agreement among the algorithms (**Figure 2**). Following the clinical interpretability and redundancy considerations, 20 variables were selected to be used in developing the models (**Table 2**).

**Table 2:** Feature selection methods and consensus results.

Screening model	Number of selected features	Selected features
LassoCV	43	Age, gender, myocardial infarction, congestive heart failure, peripheral vascular disease, dementia, cerebrovascular disease, chronic pulmonary disease, rheumatic disease, peptic ulcer disease, mild liver disease, diabetes, paraplegia, renal disease, malignant cancer, aspirin, heparin, statin, sedation, mechanical ventilation, heart rate, SBP, DBP, MBP, respiratory rate, temperature, SpO_2_, hemoglobin, platelets, WBC, anion gap, bicarbonate, BUN, chloride, creatinine, glucose, sodium, potassium, INR, PTT, lactate, PH, APSIII
mRMR	45	BUN, SpO_2_, sodium, age, APSIII, glucose, MBP, creatinine, diabetes, potassium, congestive heart failure, cerebrovascular disease, anion gap, DBP, WBC, INR, renal disease, mechanical ventilation, bicarbonate, heart rate, lactate, PH, peripheral vascular disease, chronic pulmonary disease, SBP, hemoglobin, PT, gender, paraplegia, dementia, myocardial infarction, hematocrit, mild liver disease, malignant cancer, chloride, rheumatic disease, aspirin、PTT, temperature, respiratory rate, sedation, statin, peptic ulcer disease, heparin, platelets
*F*-test	45	BUN, APSIII, creatinine, MBP, age, DBP, sodium, renal disease, potassium, glucose, anion gap, congestive heart failure, diabetes, bicarbonate, SpO_2_, lactate, PH, hemoglobin, heart rate, INR, SBP, WBC, cerebrovascular disease, hematocrit, peripheral vascular disease, PT, mechanical ventilation, gender, chronic pulmonary disease, chloride, PTT, myocardial infarct, paraplegia, temperature, aspirin, respiratory rate, sedation, mild liver disease, malignant cancer, rheumatic disease, dementia, statin, heparin, platelets, peptic ulcer disease
Mutual information	45	Creatinine, potassium, BUN, mild liver disease, WBC, SpO_2_, APSIII, chronic pulmonary disease, hemoglobin, heart rate, dementia, age, renal disease, PTT, peripheral vascular disease, MBP, glucose, diabetes, anion gap, mechanical ventilation, heparin, temperature, cerebrovascular disease, paraplegia, peptic ulcer disease, DBP, hematocrit, myocardial infarct, congestive heart failure, rheumatic disease, gender, SBP, respiratory rate, aspirin, malignant cancer, sedation, statin, chloride, bicarbonate, platelets, sodium, PT, INR, lactate, PH

### 3.3 Primary outcome: AKI prediction

Among the eight ML models evaluated for the primary outcome of AKI, the XGBoost model demonstrated the best discriminative performance. In the training set, the XGBoost model achieved an AUC of 0.787 (95% CI: 0.770–0.804); in the internal test set, it achieved an AUC of 0.659 (95% CI: 0.629–0.688), the highest among the eight algorithms (test AUCs: LightGBM 0.656, GBDT 0.650, AdaBoost 0.641, SVM 0.620, decision tree 0.612, Gaussian naive Bayes 0.606, MLP 0.606; **Figure 3A**, **Table 3**). In the test set, the XGBoost model achieved an average precision of 0.865 (95% CI: 0.861–0.869), which was superior to the other algorithms (**Figure 3B**). The validation AUC distribution across repeated cross-validation folds confirmed the stability and robustness of the XGBoost model (**Figure 4**). The sensitivity, accuracy, and F1-score of the XGBoost model at the optimal cutoff were 0.728, 0.715, and 0.796, respectively (**Table 3**). An independent re-analysis of the full model-comparison pipeline (fixed random seed, grid-search hyperparameter tuning, five-fold cross-validation) confirmed these results and showed that LightGBM, GBDT, and DT achieved distinct test-set AUCs of 0.656, 0.650, and 0.612, respectively. Pairwise DeLong tests on the test set confirmed that XGBoost significantly outperformed GBDT (*P* = 0.038), AdaBoost (*P* = 0.016), DT (*P* < 0.001), GNB (*P* < 0.001), SVM (*P* = 0.007), and MLP (*P* < 0.001), and was numerically but not significantly superior to LightGBM (*P* = 0.585). At the Youden-optimal threshold (0.758) in the test set, the XGBoost model achieved a sensitivity of 0.617 (95% CI: 0.590–0.645), specificity of 0.629 (0.580–0.677), PPV of 0.842 (0.818–0.866), and NPV of 0.339 (0.304–0.374); the corresponding metrics for all eight algorithms are reported in **Supplementary Table S7**.

**Figure 3:**
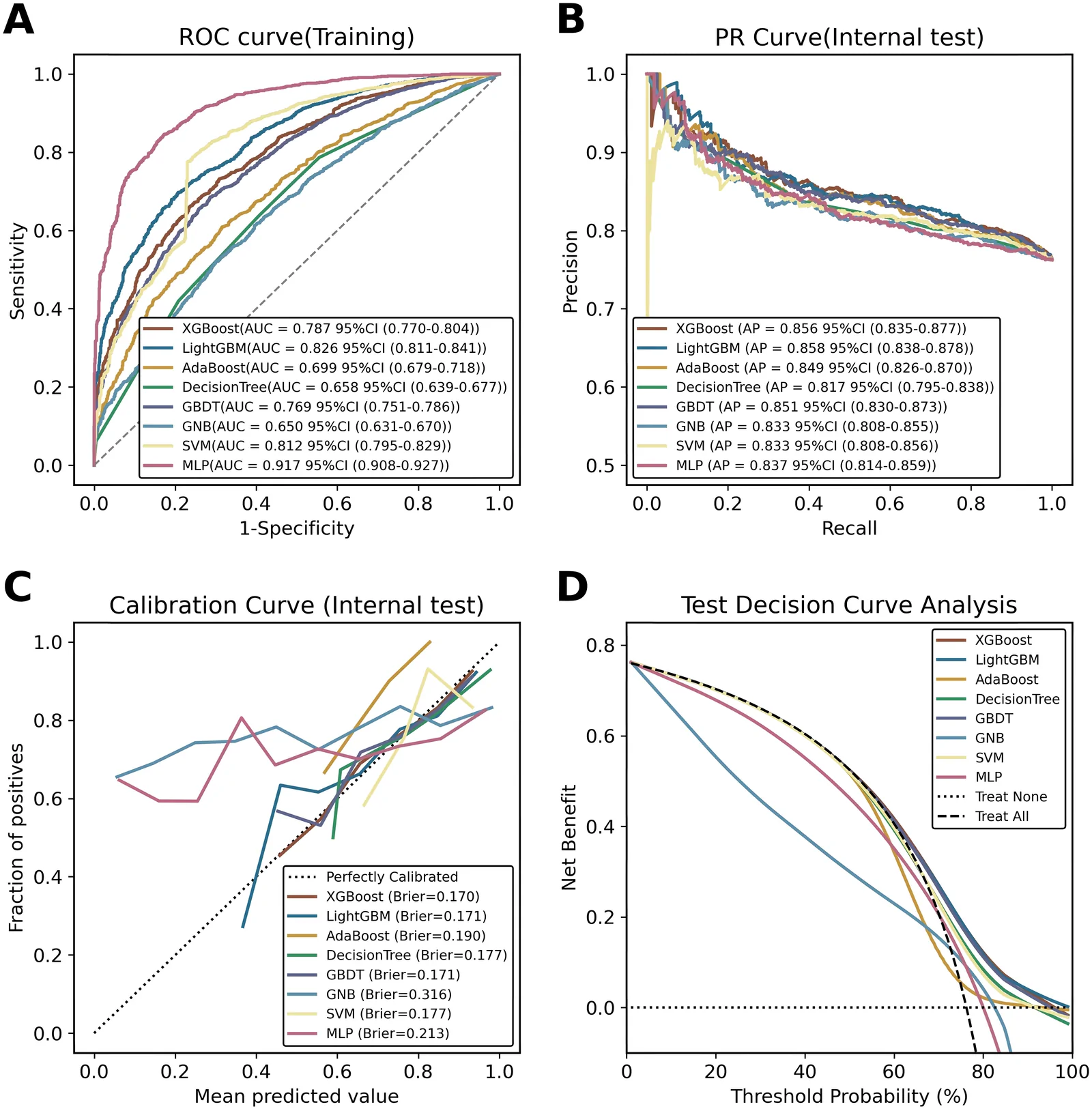
Performance of the eight machine learning models for predicting early AKI (primary outcome). **(A)** ROC curves in the training set. **(B)** Precision-recall curves in the internal test set. **(C)** Calibration curves in the internal test set. **(D)** Decision curve analysis in the internal test set.

**Table 3:** Performance comparison of eight machine learning models for AKI prediction.

Model	Training AUC (95% CI)	Internal test AUC (95% CI)	DeLong P vs. XGBoost (test)
XGBoost	0.787 (0.770–0.804)	0.659 (0.629–0.688)	Reference
LightGBM	0.826 (0.811–0.841)	0.656 (0.627–0.685)	0.585
GBDT	0.769 (0.751–0.786)	0.650 (0.621–0.680)	0.038
AdaBoost	0.699 (0.679–0.718)	0.641 (0.612–0.673)	0.016
SVM	0.812 (0.795–0.829)	0.620 (0.588–0.655)	0.007
DT	0.658 (0.639–0.677)	0.612 (0.579–0.642)	<0.001
GNB	0.650 (0.631–0.670)	0.606 (0.573–0.639)	<0.001
MLP	0.917 (0.908–0.927)	0.606 (0.574–0.639)	<0.001

Training set and internal test AUCs (95% CIs) from the verified independent re-analysis are shown, with DeLong tests versus XGBoost on the internal test set.

**Figure 4:**
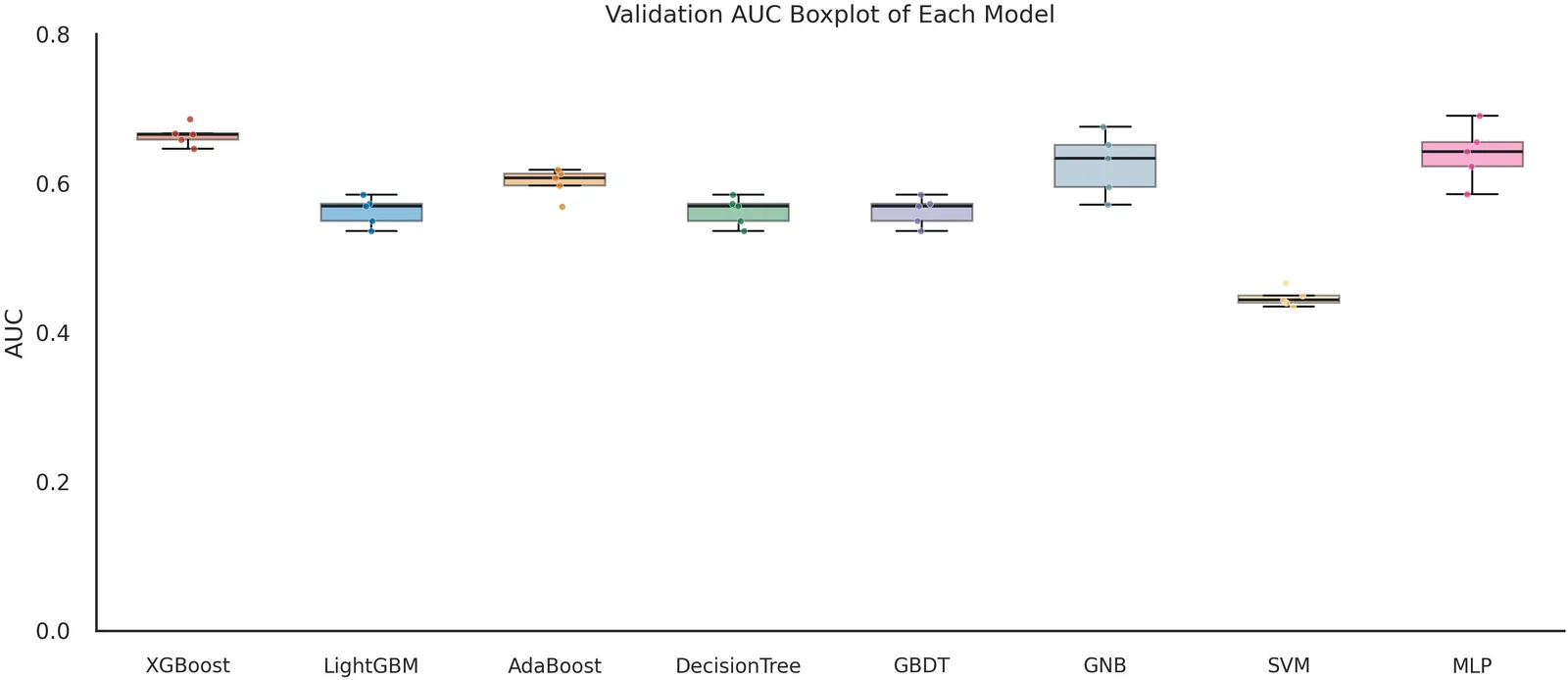
Validation AUC distribution box plot across repeated cross-validation for each model for AKI prediction.

### 3.4 Primary outcome: model calibration and clinical utility

The calibration curve of the XGBoost model in predicting AKI demonstrated a strong correlation between the predicted probability and the actual outcomes, with a Brier score of 0.170, which is also lower than the Brier scores of the other models (**Figure 3C**). The decision curve analysis found that the XGBoost model offered more net clinical benefit in comparison to the treat-all and treat-none strategies at most threshold probabilities (approx. 10% to 70%), which is a clinically desirable result (**Figure 3D**). Quantitatively, at threshold probabilities of 0.6 and 0.7, the model yielded a net benefit of 42.7 and 27.5 net true-positive equivalents per 100 patients, versus 40.6 and 20.8 for the treat-all strategy. Compared with an APSIII-only logistic reference model (AUC 0.608), the XGBoost model achieved a categorical NRI of 0.093 and an IDI of 0.047, indicating improved risk reclassification (**Supplementary Table S8**). Setting scale_pos_weight to the class-imbalance ratio (0.31) did not materially change discrimination (test AUC 0.655 vs. 0.659), so the unweighted model was retained.

### 3.5 Primary outcome: model interpretability

The SHAP analysis was used to explain the XGBoost model that predicts AKI. The five predictors with the highest mean absolute SHAP values were SpO_2_, age, APSIII score, creatinine, and white blood cell count (**Figure 5A**). The direction and magnitude of each feature contribution were represented in the SHAP summary plot by showing that higher age, lower SpO_2_, higher APSIII score, increased creatinine, and increased white blood cell count were related to higher predicted AKI risk (**Figure 5B**). Specific values of critical variables were shown in the individual force plot how they pushed the model prediction towards the base value to the final output of a representative patient (**Figure 5C**).

**Figure 5:**
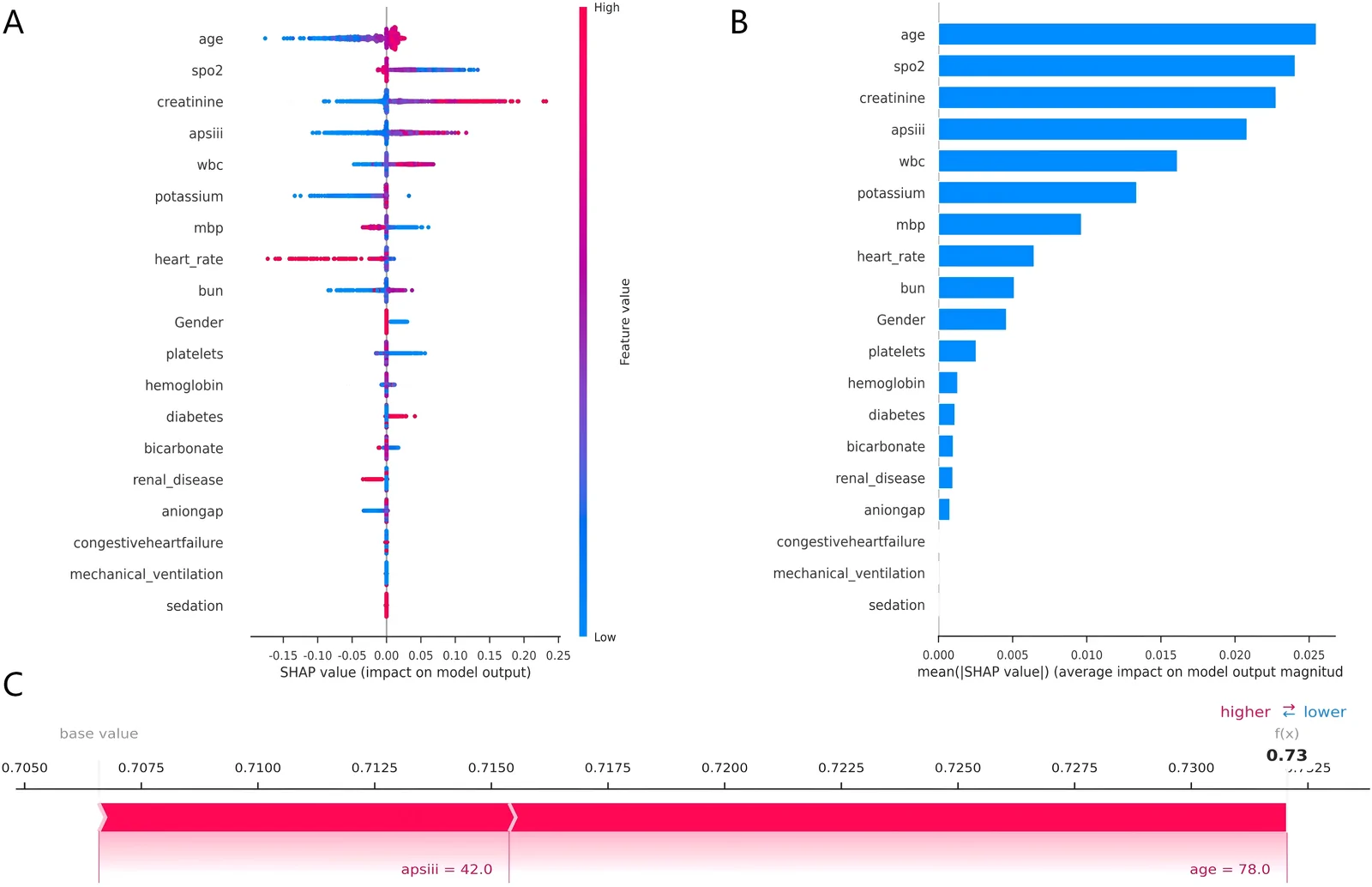
**(A)** SHAP feature importance bar plot for the XGBoost model for AKI prediction. **(B)** SHAP summary plot (beeswarm) showing the distribution of SHAP values for each feature for AKI prediction. **(C)** SHAP force plot for a representative patient, illustrating how individual variables push the prediction from the base value for AKI prediction.

### 3.6 Secondary outcome: altered consciousness prediction

The XGBoost model showed an excellent discriminative performance with respect to the secondary outcome of altered consciousness within 24 h after ICU admission. After changing the endpoint to altered consciousness (GCS ≤ 12) instead of early AKI, the XGBoost model, which was built according to the pre-specified modeling process strictly, maintained strong predictive performance (**Table 4**). The model had great discriminative ability in the test group, where it had an area under the receiver operating characteristic curve of 0.951 (95% CI: 0.936–0.963). At the Youden-optimal threshold (0.188) in the test set, sensitivity was 0.842 (95% CI: 0.804–0.879), specificity 0.932 (0.918–0.946), PPV 0.788 (0.747–0.828), and NPV 0.952 (0.939–0.964).

**Table 4:** Discriminative capacity and classification performance of the XGBoost model for predicting altered consciousness (GCS ≤ 12) within 24 h after ICU admission.

Cohort	AUC (95% CI)	Training-derived cutoff	Sensitivity (95% CI)	Specificity (95% CI)	PPV (95% CI)	NPV (95% CI)	Brier score
Internal test set	0.951 (0.936–0.963)	0.210	0.820 (0.780–0.859)	0.939 (0.926–0.953)	0.802 (0.762–0.843)	0.946 (0.933–0.958)	0.057
External validation	0.679 (0.615–0.737)	0.210	0.820 (0.773–0.866)	0.504 (0.418–0.590)	0.773 (0.724–0.822)	0.575 (0.484–0.666)	0.257

Sensitivity, specificity, PPV, and NPV with 95% CIs at the training-derived Youden threshold are reported in the internal test set and the external validation cohort; values are from the verified independent re-analysis.

The internal test ROC curve had an AUC of 0.951 (95% CI: 0.936–0.963), which is a very good predictive capability (**Figure 6A**). The calibration curve was highly consistent with the predicted probabilities and the observed outcomes, and it had a Brier score of 0.058 (95% CI: 0.048–0.067), an intercept of −0.179, a slope of 0.950, and a calibration-in-the-large (CITL) of −0.113 (**Figure 6B**). Clinical utility was also established by decision curve analysis, where the XGBoost model demonstrated significantly greater net benefits over treat-all and treat-none at a wide range of threshold probabilities (**Figure 6C**).

**Figure 6:**
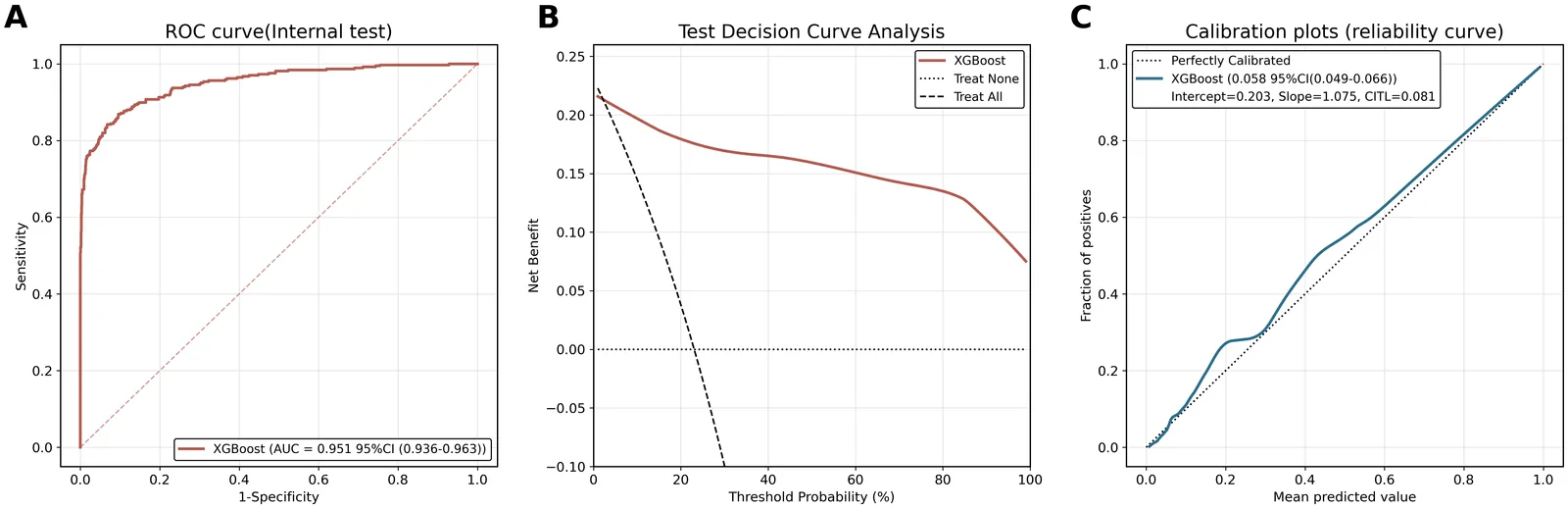
Performance of the XGBoost model for predicting altered consciousness (GCS ≤ 12) within 24 h after ICU admission (secondary outcome). **(A)** ROC curve in the internal test set. **(B)** Calibration plot in the internal test set. **(C)** Decision curve analysis in the internal test set.

In general, after replacing the endpoint with altered consciousness (GCS ≤ 12), the XGBoost model maintained its excellent discriminative performance, high prediction rate, and clinically desirable net benefit. Remarkably, the perioperative prediction model for postoperative consciousness disturbance was better in terms of discriminative ability (AUC 0.951 vs. 0.659) compared with the perioperative prediction model for AKI, which implies that perioperative variables might be more significant predictors of postoperative consciousness disturbance.

### 3.7 External validation

To further assess the generalizability of the model, this study performed external validation using data from cardiac surgery patients at Wuxi Second People’s Hospital between 1 January 2020 and 31 December 2025. The external validation cohort comprised 395 unique ICU stays after removal of duplicate records (**Figure 1**) and adopted identical inclusion and exclusion criteria, outcome definitions, and variable definitions to those of the modeling cohort; the external cohort contained no missing values, so no imputation was required for external validation. Baseline characteristics of the external cohort, alongside a comparison with the MIMIC-IV development cohort (standardized mean differences), are presented in **Supplementary Table S9**. The incidence of AKI was 28.1% (111/395), and that of altered consciousness was 67.3% (266/395); the KDIGO stage distribution of AKI events was stage 1 in 66.7%, stage 2 in 17.1%, and stage 3 in 16.2% (**Supplementary Table S5**). In the verified re-analysis, the AKI model achieved an external AUC of 0.764 (95% CI: 0.711–0.818); at the training-derived Youden threshold (0.757), sensitivity was 0.712 (95% CI: 0.627–0.796), specificity 0.736 (0.685–0.787), PPV 0.513 (0.434–0.592), and NPV 0.867 (0.824–0.910); the Brier score was 0.364, with a calibration intercept of −2.11 and a slope of 1.06, indicating systematic overestimation of absolute risk consistent with the lower AKI prevalence (28.1% vs. 76.2%). For altered consciousness, the external AUC was 0.679 (95% CI: 0.615–0.737); at the training-derived Youden threshold (0.210), sensitivity was 0.820 (0.773–0.866), specificity 0.504 (0.418–0.590), PPV 0.773 (0.724–0.822), and NPV 0.575 (0.484–0.666); the Brier score was 0.257. Calibration curves and decision curves for the external cohort are shown in **Figure 7**; **Supplementary Figures S1**, **S2**. As the external cohort contained no missing values, these results are complete data results by construction. These findings demonstrate that the model retains favorable predictive performance and generalizability in an independent external cohort, although local recalibration of the AKI model is recommended before local clinical deployment because of the substantial difference in outcome prevalence.

**Figure 7:**
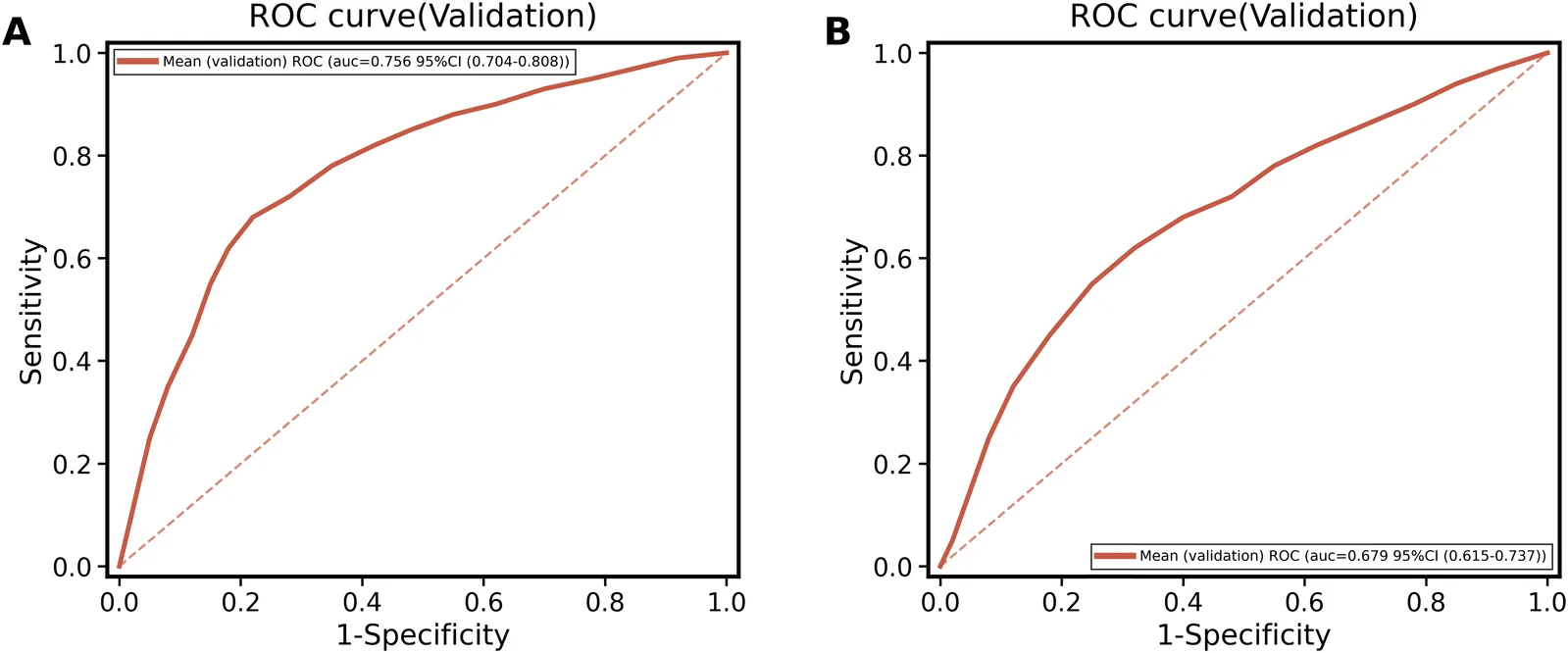
External validation of the XGBoost models in the Wuxi No.2 People’s Hospital cohort. **(A)** ROC curve for early AKI (external validation). **(B)** ROC curve for altered consciousness (external validation).

## 4 Discussion

This is a retrospective study that involved the development and validation of interpretable ML models to predict early AKI and changed consciousness risk in ICU-admitted patients undergoing cardiac surgery. Through systematic comparison between eight ML algorithms and integration of four different feature selection approaches, we found that the XGBoost model was the best predictor of both outcomes. For AKI, the model achieved an internal test AUC of 0.659 (training AUC 0.787) with acceptable calibration and clinical utility. For altered consciousness, the model performed at a better AUC of 0.951 in the internal test set, although its discrimination decreased in the external cohort (AUC 0.679). The SHAP analysis clarified the main determinants of the AKI risk, which improves the interpretability of the model and its clinical usefulness.

The early AKI rate among our cohort (76.2%) has been higher than what has been documented by other earlier research studies, and that can be explained by various reasons. To begin with, the present study involved patients admitted to the ICU following cardiac surgery, which is a more critically ill subgroup that is associated with an increased risk profile. Secondly, AKI was assessed within 48 h of ICU admission based on the KDIGO creatinine criteria that might include more early-onset cases than those based on longer periods. Thirdly, the MIMIC-IV database contains individuals who were treated at a tertiary academic medical facility and could contain a greater percentage of complicated cases (19, 20). This level of changed consciousness (22.8) was similar to the reported levels of postoperative delirium and cognitive impairment in patients undergoing cardiac surgery, which generally vary between 20% and 50% based on the diagnostic criteria and the time of evaluation (21). Notably, a high KDIGO creatinine-based AKI incidence is characteristic of ICU-managed cardiac surgery cohorts in MIMIC-IV rather than an artifact of our pipeline: Ryan et al. analyzed 4,267 cardiac surgery patients from MIMIC-IV and reported stage 1 AKI in 50%, stage 2 in 8%, and stage 3 in 4% of patients (approximately 62% any-stage AKI) (22). Our staging analysis of the external validation cohort similarly showed a predominance of stage 1 events (66.7% of AKI cases). Furthermore, sensitivity analyses in the external cohort showed that the AKI incidence estimate depends strongly on the baseline creatinine definition: 27.6% using the minimum pre-ICU value (our primary definition), 25.3% using the first ICU creatinine, and 80.5% using CKD-EPI back-calculation (**Supplementary Table S6**), confirming that our definition is intermediate rather than inflating.

In external validation, the model retained acceptable discriminative ability for AKI with an AUC of 0.764, indicating its favorable applicability across diverse critically ill cohorts. Moreover, the model yielded an AUC of 0.679 (95% CI: 0.615–0.737) in predicting postoperative altered consciousness in the external cohort, suggesting that the perioperative variables incorporated into the model may capture shared pathophysiological pathways relevant to postoperative neurological dysfunction, including hemodynamic instability, systemic inflammation, impaired organ perfusion, and disease severity. This finding underscores the broader application potential of the machine learning framework in predicting multiple complications subsequent to cardiac surgery. However, as a secondary outcome, consciousness disturbance remains exploratory in nature, and its predictive findings warrant further validation in prospective studies. Importantly, the external cohort differed markedly from the development cohort in case mix and perioperative practice (e.g., sedation exposure 30.6% vs. 96.9%, mechanical ventilation 19.7% vs. 9.0%, higher APSIII scores and haemoglobin; **Supplementary Table S9**), and the AKI prevalence was substantially lower (28.1% vs. 76.2%). Discrimination was nevertheless retained, supporting transportability of the model; however, calibration-in-the-large was poor for AKI (intercept −2.11), i.e., the model systematically overestimated absolute risk in the lower-prevalence setting. This pattern is expected when a model developed in a high-prevalence cohort is applied to a lower-prevalence population, and we therefore recommend local recalibration (intercept update, or full refitting where sample size permits) before clinical deployment. We also note that the external results reported in this revision were recomputed after removal of duplicate records from the external dataset (395 unique ICU stays).

We used a combination of the following: LassoCV, mRMR, mutual information, and *F*-test with a voting technique in our feature selection strategy to make sure that the ultimate variables were chosen based on diverse methodological points of view. This strategy minimized the probability of algorithm-specific bias and increased the resiliency of the list of features. The last 20 variables included demographics, comorbidities, vital signs, laboratory results, and severity scores, indicating that the overall evaluation of the patient condition at the time of ICU admission was performed. It is important to note that the fact that all four procedures have a significant overlap (43 common variables) indicates that the association of these variables with the outcomes is strong and repeatable under various statistical models.

The XGBoost model performed better than the other seven ML algorithms in both outcomes. In predicting AKI, the internal test AUC of 0.659 was somewhat lower than the values reported in some recent studies. Multiple prediction models of CSA-AKI were developed by Xu using MIMIC-IV, and it was found that models consisting of preoperative as well as postoperative variables had AUROC values over 0.75 (14). An XGBoost model of AKI prediction in critically ill patients with coronary heart disease was created and tested by Li et al., and it was found that the model achieved an AUROC of 0.765 in the internal test set (15). The comparatively lower test AUC in our study may be attributed to the strict leakage-free modeling pipeline (training-set-only feature selection, hyperparameter tuning, and threshold derivation) and to differences in outcome windows and case mix; notably, the model retained an AUC of 0.764 in the external validation cohort. The even larger AUC of 0.951 on altered consciousness prediction indicates that the combination of perioperative variables may prove especially useful in the identification of patients at risk of early postoperative neuropsychological complications, as altered consciousness is closer to acute physiological derangement, which is more easily measured by APSIII score and vital signs during ICU admission. This may be attributed to the fact that altered consciousness more directly reflects acute physiological derangements, whereas the APSIII score, vital signs, oxygenation status, inflammatory markers, and sedation/mechanical ventilation-related variables can all capture this acute state to a certain extent.

Our results should also be viewed in the context of recent dynamic prediction studies. Lapp et al. developed and validated a digital biomarker predicting AKI on an hourly basis up to 24 h in advance after cardiac surgery, achieving a mean AUC of 0.844 with bootstrap-aggregated regression trees (BARTm) and 0.786 with logistic regression in temporal validation (23); Ryan et al. reported an ensemble machine learning model for hourly AKI prediction in MIMIC-IV cardiac surgery patients with an AUC of 0.82 for any-stage AKI and 0.95 for stage 2 or greater AKI (22). For neurological complications, Lapp et al. achieved a mean AUC of 0.941 with support vector machines for hourly prediction of delirium onset in the ICU after cardiac surgery (24). Our admission-window XGBoost models (internal test AUC 0.659 and external AUC 0.764 for AKI; internal test AUC 0.951 for altered consciousness) require only a single set of variables from the first 24 h after ICU admission; although their AKI discrimination is lower than that of these hourly dynamic models, they provide an early, one-off risk stratification at ICU admission, whereas dynamic models support continuous risk updating, suggesting complementary roles. Differences in outcome definitions (any-stage vs. stage-specific AKI; GCS-based altered consciousness vs. CAM-ICU-diagnosed delirium), prediction windows, and study cohorts should be considered when comparing absolute performance.

The clinical applicability of SHAP interpretability analysis of the AKI model was useful. SpO_2_ became the most significant predictor, which is biologically possible because hypoxemia has been known to be a risk factor for AKI by means of renal hypoxia, oxidative stress, and inflammation (25). More advanced age and higher APSIII score, as indicators of the baseline physiological reserve and severity of acute illness, are also well-known risk factors of CSA-AKI (26, 27). Increased creatinine and white blood cell count are indicative of pre-existing renal dysfunction and systemic inflammation, respectively, and each of them is highly correlated with the development of AKI (28, 29). These SHAP effects have the same directionality as known pathophysiological mechanisms, which confirms the face validity of our model.

The predictive model established in this study may serve as a valid auxiliary tool for the early risk stratification of ICU patients following cardiac surgery. For individuals at high risk of AKI, the model can prompt clinical teams to intensify dynamic monitoring of serum creatinine, assessment of urine output, tracking of hemodynamic parameters, and management of fluid balance; promptly avoid exposure to nephrotoxic medications; and optimize individualized fluid strategies and renal protective interventions (30). In the case of patients at high risk of altered consciousness (GCS ≤ 12), the model can guide the enhancement of continuous neurological function assessment, refine adjustments to sedation and analgesia regimens, and ensure adequate oxygenation and the stability of cerebral perfusion (31). It is important to emphasize that this model is positioned as a clinical decision support system (CDSS), which is intended to assist rather than replace the professional judgment of physicians. Its value lies in facilitating the pre-emption of risk warnings and the individualization of management strategies (32).

The biological plausibility of the predictive variables is rooted in the shared pathophysiological pathways underlying kidney and central nervous system injuries after cardiac surgery. Both renal and brain tissues are highly sensitive to perioperative hypoperfusion, hypoxemia, systemic inflammation, and metabolic disorders (32). Low SpO_2_, abnormal fluctuations in blood pressure, anemia, and high disease severity scores can reflect insufficient systemic oxygen delivery and impaired organ perfusion, forming a common risk basis for AKI and delirium (33); preoperative elevated creatinine, abnormal BUN, and a history of prior kidney disease suggest a decline in renal functional reserve (33); an elevated white blood cell count reflects the activation of inflammatory responses (34); while the duration of mechanical ventilation and the use of sedative/analgesic drugs not only reflect the severity of the patient’s condition but may also directly impact neurological function assessment and cerebral metabolic homeostasis (35). By integrating hemodynamic, inflammatory, metabolic, and iatrogenic factors, these routine perioperative variables effectively capture the synergistic risk patterns of AKI and neurocognitive complications, thereby providing a theoretical basis for multi-organ protection strategies.

This study exhibits several strengths. First, the predictive model was constructed based on the large-scale public MIMIC-IV database and further subjected to external validation using the patient data from the Wuxi No.2 People’s Hospital, thereby enhancing the reliability and generalizability of the findings. Second, the integration of four feature selection methods with a voting strategy improved the stability of variable selection. Third, the model performance was systematically evaluated across three dimensions—discrimination, calibration, and clinical utility—providing a comprehensive demonstration of its clinical application value. Fourth, SHAP analysis enhanced the interpretability of the machine learning model, helping to mitigate the “black-box” limitation. Fifth, in addition to the primary outcome of AKI, this study further explored the model’s ability to predict postoperative altered mental status, expanding the potential application of this machine learning framework in risk assessment for multiple early complications following cardiac surgery.

Nevertheless, this study has several limitations. First, as a retrospective database study, despite external validation, it may still be subject to selection bias, residual confounding, and data recording bias. Prospective, multicenter studies are therefore warranted for further validation in the future. Second, the MIMIC-IV database lacks certain critical intraoperative variables, such as cardiopulmonary bypass duration, aortic cross-clamp time, duration of intraoperative hypotension, blood transfusion volume, and dosages of vasoactive agents—all of which may influence CSA-AKI and postoperative neurological outcomes. Third, AKI in this study was primarily defined based on creatinine criteria, and the completeness and accuracy of urine output data may be limited, potentially leading to underestimation or overestimation of some AKI events. Fourth, although the KNN method was used to handle missing data, data imputation may still introduce uncertainty; in the MIMIC-IV cohort, however, pre-imputation missingness was low (≤2.3% for all modeling variables; temperature, the only variable with higher missingness at 16.5%, was not retained in the final model), a complete-case sensitivity analysis yielded consistent results, and the external validation cohort contained no missing values, so residual bias from imputation is likely to be small. Fifth, the AKI model showed calibration drift in the external cohort (systematic risk overestimation) owing to the large prevalence difference, so local recalibration is required before local use. Sixth, altered consciousness defined by a GCS score ≤12 does not equate to delirium or cognitive dysfunction: RASS/SAS sedation scores were not systematically available, deeply sedated or mechanically ventilated patients could not be excluded, and the outcome captures a decreased level of consciousness of any cause rather than CAM-ICU-diagnosed delirium. Finally, while SHAP analysis provides both global and local interpretations of the model, this is an observational study and cannot be used to infer causal relationships between predictive variables and outcomes.

## 5 Conclusions

This study developed and validated an interpretable machine learning model to predict early AKI and altered consciousness (GCS ≤ 12) in patients admitted to the ICU following cardiac surgery. The XGBoost model showed moderate discrimination for early AKI (internal test AUC 0.659; external AUC 0.764) and good internal discrimination for altered consciousness (internal test AUC 0.951), although its performance decreased in the external cohort (AUC 0.679); moreover, the AKI model systematically overestimated absolute risk in the lower-prevalence external population, so local recalibration is required before any clinical use. SHAP analysis identified clinically meaningful predictors, including SpO_2_, age, APSIII, serum creatinine level, and white blood cell count, thereby enhancing the model’s interpretability. These results suggest that interpretable machine learning holds promise for facilitating early risk stratification and personalized postoperative management of cardiac surgery ICU patients. However, further prospective validation is required prior to its clinical application.

## Data Availability

The original contributions presented in the study are included in the article/**Supplementary Material**. Further inquiries can be directed to the corresponding authors.
