# Supplementary material for: Development and validation of an interpretable machine learning model for predicting early acute kidney injury and altered consciousness in cardiac surgery patients admitted to the intensive care unit: a retrospective study
Source: Front Endocrinol (Lausanne). 2026 Oct 7;17:1959304. doi: 10.3389/fendo.2026.1959304 (PMC13682982; doi:10.3389/fendo.2026.1959304)
Supplement: Supplementary file 1 [file Table1.docx]

Supplementary Material

# 1 Supplementary Figures and Tables

## 1.1 Supplementary Figures

**Supplementary Figure 1.** Performance of the XGBoost model for early AKI in the training, internal test and external validation cohorts. (A/E/I) ROC curves; (B/F/J) precision-recall curves; (C/G/K) calibration plots; (D/H/L) decision curve analyses.

**Supplementary Figure 2.** Performance of the XGBoost model for altered consciousness (GCS ≤ 12) in the training, internal test and external validation cohorts. (A/E/I) ROC curves; (B/F/J) precision-recall curves; (C/G/K) calibration plots; (D/H/L) decision curve analyses.

## 1.2 Supplementary Tables

**Supplementary Table S1.** Pre-imputation missingness rates of the 20 modeling variables in the MIMIC-IV development cohort and the external validation cohort

| **Variable** | **MIMIC-IV missingness, % (n = 5,288)** | **Wuxi missingness, % (n = 395)** |
| --- | --- | --- |
| Heart rate | 0.1 | 0.0 |
| SpO2 | 0.1 | 0.0 |
| Systolic blood pressure | 0.4 | 0.0 |
| Diastolic blood pressure | 0.4 | 0.0 |
| Mean blood pressure | 0.1 | 0.0 |
| Hemoglobin | 0.1 | 0.0 |
| Platelets | 0.1 | 0.0 |
| White blood cell count | 0.1 | 0.0 |
| Creatinine | 0.1 | 0.0 |
| Anion gap | 0.8 | 0.0 |
| Bicarbonate | 0.1 | 0.0 |
| Blood urea nitrogen | 0.1 | 0.0 |
| Potassium | 0.6 | 0.0 |
| Lactate | 0.2 | 0.0 |
| APSIII score | 0.0 | 0.0 |

**Supplementary Table S2.** Sensitivity analysis of the feature-selection voting threshold (AKI outcome, XGBoost)

| **Voting threshold** | **Features retained** | **Training CV AUC (5-fold)** | **Test-set AUC** | **Features** |
| --- | --- | --- | --- | --- |
| ≥1 | 20 | 0.679 | 0.659 | age, Gender, diabetes, renal_disease, chf, creatinine, bun, potassium, bicarbonate, aniongap, lactate, mbp, spo2, heart_rate, mv, apsiii, hemoglobin, wbc, platelets, sedation |
| ≥2 | 20 | 0.679 | 0.659 | age, Gender, diabetes, renal_disease, chf, creatinine, bun, potassium, bicarbonate, aniongap, lactate, mbp, spo2, heart_rate, mv, apsiii, hemoglobin, wbc, platelets, sedation |
| ≥3 | 18 | 0.682 | 0.659 | age, Gender, diabetes, renal_disease, chf, creatinine, bun, potassium, aniongap, lactate, mbp, spo2, heart_rate, mv, apsiii, hemoglobin, wbc, sedation |
| ≥4 | 14 | 0.676 | 0.659 | age, diabetes, chf, creatinine, bun, potassium, aniongap, lactate, mbp, spo2, heart_rate, mv, apsiii, wbc |

**Supplementary Table S3.** Feature-distribution consistency between training and test sets (maximum standardized mean difference = 0.067)

| **Variable** | **Training set (mean or proportion)** | **Test set (mean or proportion)** | **\|SMD\| / \|difference\|** |
| --- | --- | --- | --- |
| age | 67.81 | 67.8 | 0.001 |
| Gender | 0.708 | 0.729 | 0.021 |
| diabetes | 0.362 | 0.347 | 0.015 |
| renal_disease | 0.169 | 0.162 | 0.007 |
| chf | 0.263 | 0.266 | 0.003 |
| creatinine | 1.11 | 1.1 | 0.012 |
| bun | 19.23 | 19.27 | 0.004 |
| potassium | 4.65 | 4.65 | 0.002 |
| bicarbonate | 22.36 | 22.45 | 0.043 |
| aniongap | 12.81 | 12.63 | 0.067 |
| lactate | 2.93 | 2.86 | 0.052 |
| mbp | 73.84 | 74.04 | 0.035 |
| spo2 | 97.85 | 97.86 | 0.008 |
| heart_rate | 82.13 | 81.9 | 0.025 |
| mv | 0.088 | 0.096 | 0.008 |
| apsiii | 41.46 | 40.59 | 0.043 |
| hemoglobin | 9.07 | 9.14 | 0.045 |
| wbc | 16.29 | 16.79 | 0.06 |
| platelets | 134.88 | 134.97 | 0.002 |
| sedation | 0.968 | 0.97 | 0.002 |

**Supplementary Table S4.** Baseline characteristics of patients with vs without altered consciousness (GCS ≤12) in the MIMIC-IV cohort

| **Variable** | **Altered consciousness (n=1221)** | **No altered consciousness (n=4067)** | **P value** |
| --- | --- | --- | --- |
| Age, years | 70.0 (61.0-78.0) | 68.0 (60.0-76.0) | 0.002 |
| Male, n (%) | 830 (68.0) | 2949 (72.5) | 0.002 |
| Diabetes, n (%) | 452 (37.0) | 1438 (35.4) | 0.304 |
| Renal disease, n (%) | 245 (20.1) | 637 (15.7) | <0.001 |
| Congestive heart failure, n (%) | 384 (31.4) | 1012 (24.9) | <0.001 |
| Creatinine, mg/dL | 1.0 (0.8-1.3) | 0.9 (0.8-1.1) | <0.001 |
| Blood urea nitrogen, mg/dL | 18.0 (14.0-23.0) | 17.0 (14.0-21.0) | <0.001 |
| Potassium, mmol/L | 4.6 (4.3-4.9) | 4.6 (4.3-4.9) | 0.06 |
| Bicarbonate, mmol/L | 22.0 (21.0-24.0) | 22.0 (21.0-24.0) | 0.018 |
| Anion gap, mmol/L | 13.0 (11.0-15.0) | 12.0 (11.0-14.0) | <0.001 |
| Lactate, mmol/L | 2.8 (2.1-3.8) | 2.5 (2.0-3.2) | <0.001 |
| Mean blood pressure, mmHg | 73.8 (69.9-77.3) | 73.8 (70.2-77.5) | 0.646 |
| SpO2, % | 98.0 (97.0-98.9) | 98.0 (97.0-98.8) | 0.907 |
| Heart rate, bpm | 81.6 (76.7-88.4) | 81.1 (76.1-87.1) | 0.004 |
| Mechanical ventilation, n (%) | 183 (15.0) | 293 (7.2) | <0.001 |
| APSIII score | 72.0 (53.0-82.0) | 32.0 (25.0-39.0) | <0.001 |
| Hemoglobin, g/dL | 8.8 (7.8-10.0) | 9.1 (8.0-10.1) | <0.001 |
| White blood cell count, 10^9^/L | 15.5 (12.1-19.2) | 15.3 (12.3-19.3) | 0.895 |
| Platelets, 10^9^/L | 126.0 (100.0-159.0) | 130.0 (104.0-159.0) | 0.019 |
| Sedation, n (%) | 1201 (98.4) | 3922 (96.4) | <0.001 |

**Supplementary Table S5.** KDIGO stage distribution of early AKI (creatinine criteria, within 48 h after ICU admission) in the development and external validation cohorts

| **KDIGO stage** | **MIMIC-IV, n (%) (N = 5,288)** | **Wuxi, n (%) (N = 395)** |
| --- | --- | --- |
| No AKI | 1,257 (23.8) | 277 (70.1) |
| Stage 1 | 1,548 (29.3) | 74 (18.7) |
| Stage 2 | 2,192 (41.5) | 19 (4.8) |
| Stage 3 | 291 (5.5) | 25 (6.3) |
| Among AKI events: stage 1 / 2 / 3, % | 38.4 / 54.4 / 7.2 | 66.7 / 17.1 / 16.2 |

**Supplementary Table S6.** Sensitivity of AKI incidence to the baseline creatinine definition (external validation cohort, n = 395)

| **Baseline creatinine definition** | **AKI incidence in external validation cohort, %** |
| --- | --- |
| Baseline min preICU (original) | 27.6 |
| Baseline first ICU_creatinine | 25.3 |
| Baseline CKD-EPI backcalc_eGFR75 | 80.5 |

**Supplementary Table S7.** Classification performance of all eight machine learning models at the Youden-optimal threshold (internal test set)

| **Outcome** | **Model** | **Test AUC (95% CI)** | **Cutoff** | **Sensitivity (95% CI)** | **Specificity (95% CI)** | **PPV (95% CI)** | **NPV (95% CI)** |
| --- | --- | --- | --- | --- | --- | --- | --- |
| AKI | XGBoost | 0.659 (0.629-0.688) | 0.757 | 0.617 (0.59-0.645) | 0.626 (0.577-0.675) | 0.841 (0.817-0.865) | 0.338 (0.303-0.373) |
| AKI | LightGBM | 0.656 (0.627-0.685) | 0.754 | 0.631 (0.604-0.659) | 0.589 (0.539-0.639) | 0.831 (0.807-0.856) | 0.332 (0.297-0.368) |
| AKI | GBDT | 0.65 (0.621-0.68) | 0.752 | 0.632 (0.605-0.659) | 0.581 (0.531-0.631) | 0.829 (0.805-0.853) | 0.33 (0.294-0.366) |
| AKI | AdaBoost | 0.641 (0.612-0.673) | 0.626 | 0.579 (0.551-0.606) | 0.626 (0.577-0.675) | 0.832 (0.807-0.858) | 0.316 (0.283-0.35) |
| AKI | DT | 0.612 (0.579-0.642) | 0.764 | 0.707 (0.681-0.732) | 0.44 (0.39-0.49) | 0.802 (0.778-0.826) | 0.319 (0.279-0.359) |
| AKI | GNB | 0.606 (0.573-0.639) | 0.551 | 0.474 (0.446-0.503) | 0.658 (0.61-0.706) | 0.817 (0.788-0.845) | 0.281 (0.251-0.31) |
| AKI | SVM | 0.62 (0.588-0.655) | 0.767 | 0.56 (0.532-0.587) | 0.599 (0.55-0.649) | 0.818 (0.791-0.844) | 0.298 (0.265-0.33) |
| AKI | MLP | 0.606 (0.574-0.639) | 0.712 | 0.7 (0.674-0.726) | 0.422 (0.372-0.472) | 0.795 (0.771-0.82) | 0.305 (0.265-0.344) |
| Altered consciousness | XGBoost | 0.951 (0.936-0.963) | 0.21 | 0.82 (0.78-0.859) | 0.939 (0.926-0.953) | 0.802 (0.762-0.843) | 0.946 (0.933-0.958) |
| Altered consciousness | LightGBM | 0.944 (0.927-0.958) | 0.214 | 0.806 (0.765-0.847) | 0.944 (0.931-0.957) | 0.813 (0.773-0.853) | 0.942 (0.929-0.955) |
| Altered consciousness | GBDT | 0.946 (0.929-0.959) | 0.18 | 0.863 (0.828-0.899) | 0.915 (0.899-0.93) | 0.752 (0.711-0.794) | 0.957 (0.946-0.969) |
| Altered consciousness | AdaBoost | 0.947 (0.931-0.96) | 0.436 | 0.861 (0.825-0.896) | 0.9 (0.883-0.917) | 0.721 (0.679-0.763) | 0.956 (0.944-0.968) |
| Altered consciousness | DT | 0.936 (0.917-0.951) | 0.25 | 0.842 (0.804-0.879) | 0.918 (0.903-0.933) | 0.755 (0.713-0.797) | 0.951 (0.938-0.963) |
| Altered consciousness | GNB | 0.893 (0.871-0.913) | 0.461 | 0.749 (0.704-0.793) | 0.907 (0.891-0.924) | 0.708 (0.663-0.753) | 0.923 (0.908-0.938) |
| Altered consciousness | SVM | 0.95 (0.935-0.964) | 0.318 | 0.795 (0.754-0.836) | 0.957 (0.946-0.969) | 0.848 (0.81-0.886) | 0.94 (0.926-0.953) |
| Altered consciousness | MLP | 0.92 (0.901-0.939) | 0.264 | 0.792 (0.751-0.834) | 0.916 (0.9-0.931) | 0.738 (0.694-0.781) | 0.936 (0.923-0.95) |

Decision thresholds are now derived by maximizing the Youden index on the training set and applied unchanged to the test set.

**Supplementary Table S8a.** Reclassification metrics (NRI/IDI) of XGBoost vs an APSIII-only reference model (internal test set)

| **Outcome** | **Reference model** | **NRI (events)** | **NRI (non-events)** | **NRI (total)** | **IDI** |
| --- | --- | --- | --- | --- | --- |
| AKI | APSIII-only logistic regression (AUC 0.608) | 0.186 | -0.093 | 0.093 | 0.047 |
| Altered consciousness | APSIII-only logistic regression (AUC 0.939) | -0.011 | 0.02 | 0.01 | 0.032 |

**Supplementary Table S8b.** Absolute net benefit per 100 patients of the XGBoost models vs treat-all (internal test set)

| **Threshold probability** | **AKI: model net benefit /100** | **AKI: treat-all /100** | **Altered consciousness: model /100** | **Altered consciousness: treat-all /100** |
| --- | --- | --- | --- | --- |
| 0.1 | 73.6 | 73.6 | 19.42 | 14.51 |
| 0.2 | 70.31 | 70.31 | 17.83 | 3.83 |
| 0.3 | 66.06 | 66.06 | 16.78 | -9.91 |
| 0.4 | 60.53 | 60.41 | 16.45 | -28.23 |
| 0.5 | 52.87 | 52.49 | 16.07 | -53.88 |
| 0.6 | 42.66 | 40.61 | 15.15 | -92.34 |
| 0.7 | 27.52 | 20.81 | 14.2 | -156.46 |

**Supplementary Table S9.** Baseline characteristics of the MIMIC-IV development cohort and the external validation cohort

| **Variable** | **MIMIC-IV (n=5288)** | **external validation cohort (n=395)** | **SMD** |
| --- | --- | --- | --- |
| Age, years | 68.0 (60.0-76.0) | 66.0 (57.0-75.0) | 0.269 |
| Male, n (%) | 3779 (71.5) | 255 (64.6) | 0.148 |
| Diabetes, n (%) | 1890 (35.7) | 133 (33.7) | 0.044 |
| Renal disease, n (%) | 882 (16.7) | 59 (14.9) | 0.048 |
| Congestive heart failure, n (%) | 1396 (26.4) | 85 (21.5) | 0.115 |
| Creatinine, mg/dL | 0.9 (0.8-1.2) | 1.0 (0.8-1.3) | 0.202 |
| Blood urea nitrogen, mg/dL | 17.0 (14.0-22.0) | 15.0 (11.0-21.0) | 0.079 |
| Potassium, mmol/L | 4.6 (4.3-4.9) | 4.2 (3.8-4.6) | 0.75 |
| Bicarbonate, mmol/L | 22.0 (21.0-24.0) | 23.0 (21.0-25.0) | 0.326 |
| Anion gap, mmol/L | 13.0 (11.0-14.0) | 8.0 (6.0-10.9) | 1.175 |
| Lactate, mmol/L | 2.6 (2.0-3.4) | 1.8 (1.3-3.1) | 0.05 |
| Mean blood pressure, mmHg | 73.8 (70.1-77.4) | 79.4 (74.3-86.6) | 0.829 |
| SpO2, % | 98.0 (97.0-98.8) | 98.0 (96.0-99.1) | 0.307 |
| Heart rate, bpm | 81.2 (76.2-87.4) | 85.0 (77.6-92.4) | 0.306 |
| Mechanical ventilation, n (%) | 476 (9.0) | 78 (19.7) | 0.31 |
| APSIII score | 35.0 (27.0-48.0) | 58.0 (40.0-75.0) | 0.781 |
| Hemoglobin, g/dL | 9.0 (8.0-10.1) | 10.7 (9.4-12.3) | 0.988 |
| White blood cell count, 10^9^/L | 15.4 (12.2-19.3) | 12.3 (9.5-16.4) | 0.413 |
| Platelets, 10^9^/L | 129.0 (103.0-159.0) | 142.0 (108.5-189.5) | 0.304 |
| Sedation, n (%) | 5123 (96.9) | 121 (30.6) | 1.902 |
